# Saikosaponin A Recovers Impaired Filaggrin Levels in Inflamed Skin by Downregulating the Expression of FRA1 and c-Jun

**DOI:** 10.3390/molecules29174064

**Published:** 2024-08-27

**Authors:** Sung Shin Ahn, Hyunjin Yeo, Euitaek Jung, Tae Yoon Kim, Junekyu Han, Young Han Lee, Soon Young Shin

**Affiliations:** 1 Department of Biological Sciences, Sanghuh College of Lifesciences, Konkuk University, Seoul 05029, Republic of Korea; wendy713@konkuk.ac.kr (S.S.A.); jini1606@konkuk.ac.kr (H.Y.); mylife4sci@konkuk.ac.kr (E.J.); yun0614@konkuk.ac.kr (T.Y.K.); wnsrb0422@konkuk.ac.kr (J.H.); yhlee58@konkuk.ac.kr (Y.H.L.); 2 Cancer and Metabolism Institute, Konkuk University, Seoul 05029, Republic of Korea

**Keywords:** saikosaponin A, atopic dermatitis, filaggrin, FOS-like antigen protein 1, c-Jun, extracellular signal-regulated kinase

## Abstract

Filaggrin (FLG) is an essential structural protein expressed in differentiated keratinocytes. Insufficient FLG expression contributes to the pathogenesis of chronic inflammatory skin diseases. Saikosaponin A (SSA), a bioactive oleanane-type triterpenoid, exerts anti-inflammatory activity. However, the effects of topically applied SSA on FLG expression in inflamed skin remain unclear. This study aimed to evaluate the biological activity of SSA in restoring reduced FLG expression. The effect of SSA on FLG expression in HaCaT cells was assessed through various biological methods, including reverse transcription PCR, quantitative real-time PCR, immunoblotting, and immunofluorescence staining. TNFα and IFNγ decreased FLG mRNA, cytoplasmic FLG protein levels, and FLG gene promoter–reporter activity compared to the control groups. However, the presence of SSA restored these effects. A series of FLG promoter–reporter constructs were generated to investigate the underlying mechanism of the effect of SSA on FLG expression. Mutation of the AP1-binding site (mtAP1) in the −343/+25 FLG promoter–reporter abrogated the decrease in reporter activities caused by TNFα + IFNγ, suggesting the importance of the AP1-binding site in reducing FLG expression. The SSA treatment restored FLG expression by inhibiting the expression and nuclear localization of FRA1 and c-Jun, components of AP1, triggered by TNFα + IFNγ stimulation. The ERK1/2 mitogen-activated protein kinase signaling pathway upregulates FRA1 and c-Jun expression, thereby reducing FLG levels. The SSA treatment inhibited ERK1/2 activation caused by TNFα + IFNγ stimulation and reduced the levels of FRA1 and c-Jun proteins in the nucleus, leading to a decrease in the binding of FRA1, c-Jun, p-STAT1, and HDAC1 to the AP1-binding site in the FLG promoter. The effect of SSA was evaluated in an animal study using a BALB/c mouse model, which induces human atopic-dermatitis-like skin lesions via the topical application of dinitrochlorobenzene. Topically applied SSA significantly reduced skin thickening, immune cell infiltration, and the expression of FRA1, c-Jun, and p-ERK1/2 compared to the vehicle-treated group. These results suggest that SSA can effectively recover impaired FLG levels in inflamed skin by preventing the formation of the repressor complex consisting of FRA1, c-Jun, HDAC1, and STAT1.

## 1. Introduction

The epidermis, which serves as the outermost layer of the skin, primarily consists of keratinocytes that undergo differentiation and migration toward the surface of the skin [1,2]. Filaggrin (FLG; filament aggregating protein), a filament-associated protein that binds to keratin fibers, plays a crucial function in preserving the structural integrity of the skin barrier [3]. FLG is initially expressed as a large precursor protein (>400 kDa) called proFLG, which is degraded into 10–12 FLG monomers (>37 kDa each) in differentiating keratinocytes [4]. Monomeric FLG binds to keratin filaments and promotes keratinocyte flattening, preventing water loss and external pathogen invasion, suggesting that FLG contributes to stratum corneum integrity and skin barrier function [5].

Loss of FLG expression in keratinocytes can disrupt the skin barrier, compromising its protective function [6]. Genetic problems associated with FLG, such as loss-of-function mutations or truncated proteins, have been reported in many patients with atopic dermatitis (AD) and ichthyosis vulgaris [7,8]. FLG expression is reduced in keratinocytes treated with proinflammatory cytokines and in skin lesions of patients with AD, suggesting that a characteristic of chronic inflammatory skin diseases is the reduced expression of FLG, an established genetic factor of chronic skin inflammation [9]. During pathogenesis, multiple inflammatory cells, including lymphocytes and mast cells, release various inflammatory cytokines and chemokines into the dermis and epidermis [10]. Patients with inflammatory skin diseases exhibit elevated levels of tumor necrosis factor α (TNFα) and interferon γ (IFNγ) in their serum [11]. Additionally, TNFα and IFNγ increase the expression of other inflammatory cytokines, including interleukin-17 (IL-17) and thymic stromal lymphopoietin (TSLP) [11], and disrupt the skin barrier via inhibition of the expression of FLG [12]. Our previous study demonstrated that TNFα + IFNγ treatment reduced FLG expression in human keratinocytes and skin lesions of mouse models with 2,4-dinitrochlorobenzene (DNCB)-induced AD-like and imiquimod-induced psoriasis-like symptoms [13]. These findings suggest that FLG expression is reduced via inflammatory cytokines such as TNFα and IFNγ.

Numerous natural substances have been used to alleviate skin inflammation and enhance skin barrier functions [14]. Radix Bupleuri, a dried root of *Bupleurum* species, is a natural product used in oriental folk medicine [15,16]. Saikosaponins are bioactive oleanane-type triterpenoid saponins isolated from Radix Bupleuri [17]. They have various beneficial effects, including antiviral, anticancer, immunomodulatory, and anti-inflammatory activities [17,18]. Saikosaponin A (SSA) and saikosaponin C (SSC) ameliorated AD-like skin lesions by reducing TSLP expression [13]; however, the impact of SSA on FLG expression in inflamed skin remains unclear.

The TNFα + IFNγ-induced reduction in FLG expression is mediated by binding the histone deacetylase (HDAC) repressor complex to the FLG promoter [12]. This study aimed to evaluate the impact of SSA on restoring impaired FLG expression in TNFα + IFNγ-stimulated HaCaT cells, an immortalized human keratinocyte cell line, and in a DNCB-induced AD-like BALB/c mouse model. The results demonstrated that SSA effectively restored impaired FLG levels in TNFα + IFNγ-stimulated HaCaT cells by inhibiting ERK1/2 activation and reducing the levels of FRA1 and c-Jun proteins in the nucleus, leading to a decrease in the formation of the repressor complex consisting of FRA1, c-Jun, p-STAT1, and HDAC1 to the AP1-binding site in the FLG promoter. In addition, in vitro findings were confirmed by in vivo animal experiments using a BALB/c mouse model, which induces human AD-like skin lesions via the topical application of DNCB.

## 2. Results

### 2.1. Effect of SSA on Cytotoxicity in HaCaT Keratinocytes

**Figure 1:**
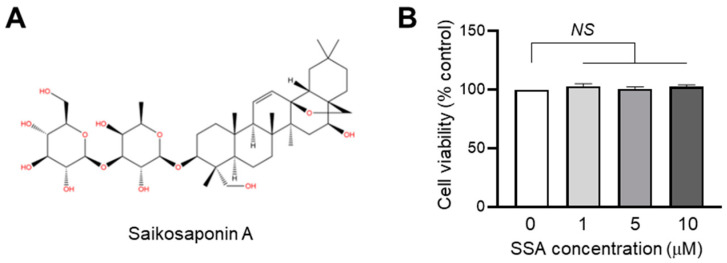
An examination of the cytotoxic effects of SSA. (**A**) Chemical structure of SSA. (**B**) HaCaT cells were exposed to varying concentrations of SSA for 24 h. Cell viabilities were measured using the Cell Counting Kit-8 (CCK-8). Bars indicate the mean ± S.D. (*n* = 3). NS = no significance, ^NS^ *p* = 0.1941 (0 vs. 1 μM), ^NS^ *p* = 0.6947 (0 vs. 5 μM), ^NS^ *p* = 0.1491 (0 vs. 10 μM) by Sidak’s multiple comparisons test.

Before determining the effect of SSA (Figure 1A) on FLG expression, a CCK-8 assay was performed using water-soluble formazan to evaluate the cytotoxic effect of SSA on HaCaT cells (Figure 1B). SSA was not cytotoxic at concentrations up to 10 μM in HaCaT keratinocytes. Therefore, in the following experiments, a non-cytotoxic SSA concentration (1 μM) was selected.

### 2.2. Inhibitory Effect of SSA on TNFα + IFNγ-Induced Suppression of FLG Expression in HaCaT Keratinocytes

**Figure 2:**
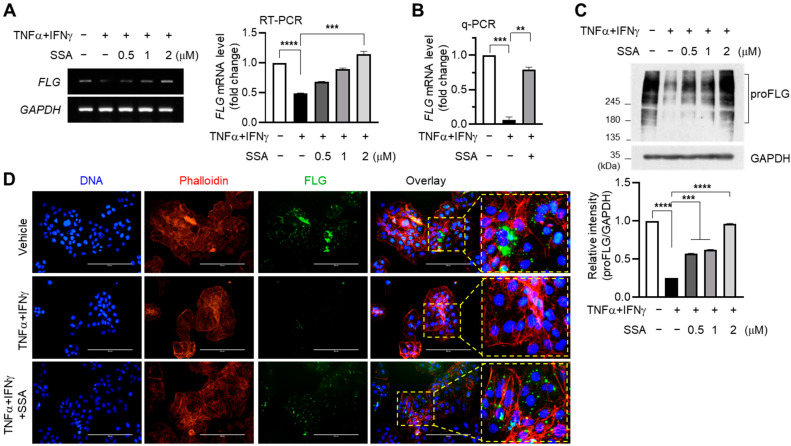
Restoration effect of SSA on the TNFα + IFNγ-induced suppression of FLG expression in HaCaT cells. HaCaT cells pre-exposed to SSA for 30 min were treated with TNFα + IFNγ (10 ng/mL). (**A**) After 24 h, mRNA levels of FLG and GAPDH were measured using RT-PCR. Relative FLG mRNA levels were measured using ImageJ version 1.52a software. (**B**) Quantitative real-time PCR (q-PCR). (**C**) proFLG protein levels were examined using immunoblotting. The band intensity corresponding to the FLG protein level was normalized to the GAPDH level using ImageJ version 1.52a software. The data are displayed as mean ± SD (*n* = 3). ** *p* < 0.01, *** *p* < 0.001, **** *p* < 0.0001. (**D**) HaCaT cells seeded on coverslips were treated with TNFα + IFNγ (10 ng/mL) for 24 h with or without 1 μM SSA, and immunofluorescence staining was carried out with a proFLG antibody. Nuclear DNA was counterstained using Hoechst 33258. Scale bars, 200 μm. RT-PCR, reverse transcription polymerase chain reaction; q-PCR, quantitative real-time PCR; SSA, saikosaponin A.

TNFα and IFNγ are proinflammatory cytokines that promote the expression of various other inflammatory cytokines and chemokines [5,19]. Reverse transcription PCR (RT-PCR) data revealed that the TNFα + IFNγ-induced decrease in FLG mRNA levels was recovered via SSA pretreatment in a dose-dependent manner (Figure 2A). A quantitative real-time PCR (q-PCR) analysis showed that TNFα and IFNγ inhibited FLG mRNA levels compared to those of the control, which was significantly (*p* < 0.001) increased by pretreatment with 1 µM SSA (Figure 2B). Notably, the level of FLG mRNA is linearly dependent on SSA concentration without any saturation even after it exceeds the control level. This suggests that SSA could directly impact the activation of certain kinases like ERK, JNK, and p38 kinases directly rather than through indirect effects.

The immunoblot analysis showed that pretreatment with SSA effectively abrogated the TNFα + IFNγ-mediated FLG downregulation in protein levels (Figure 2C). Fluorescence immunocytochemical staining showed that TNFα + IFNγ treatment reduced the cytoplasmic localization of FLG, which was significantly recovered in the presence of SSA (Figure 2D). These data suggest that SSA can restore TNFα + IFNγ-induced FLG suppression in mRNA and protein levels in HaCaT cells.

### 2.3. SSA Inhibits TNFα + IFNγ-Induced Repression of FLG Transcription

**Figure 3:**
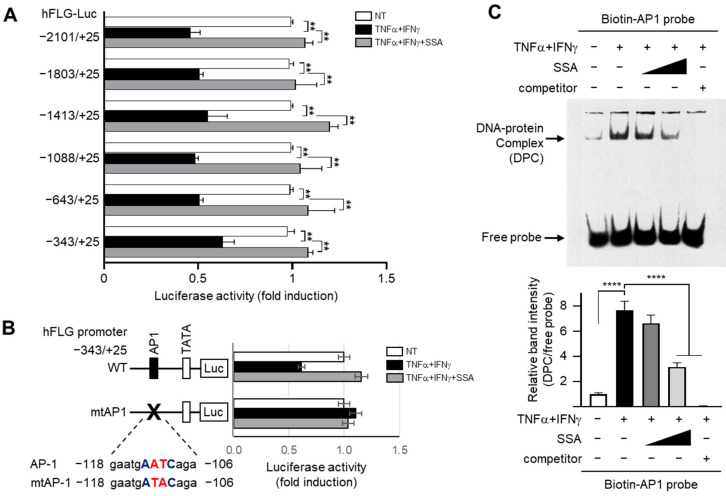
Effect of SSA on the inhibition of TNFα + IFNγ-induced suppression of FLG transcription. (**A**) HaCaT cells transfected with 0.2 μg of a series of 5′-deletion constructs of the FLG promoter were stimulated with TNFα + IFNγ (10 ng/mL) with or without 1 μM SSA. After 8 h, luciferase promoter–reporter activities were examined. (**B**) HaCaT cells were transfected with either the wild-type (WT) or mutant version of the plasmids carrying a point mutation in the AP1-binding sequence (−118/−106) of the pFLG-Luc plasmid (mtAP-1). Following a 24 h incubation, the cells were exposed to TNFα + IFNγ for 8 h, and luciferase activities were measured. (**C**) HaCaT cells were treated with TNFα + IFNγ in the presence or absence of 1 μM SSA. After 24 h, nuclear extracts (3 μg) were incubated with a biotinylated AP1-binding oligonucleotide probe (50 fmol) in the presence or absence of an unlabeled probe (competitor, 2500 fmol), and the EMSA analysis was performed. Relative band intensity was measured using ImageJ version 1.52a software. The data are displayed as mean ± SD (*n* = 3). **p < 0.01. **** *p* < 0.0001. AP-1, activator protein 1; mtAP-1, mutation of the AP-1; SSA, saikosaponin A.

The luciferase assay data showed that TNFα + IFNγ significantly (all *p* < 0.01) decreased luciferase reporter activity, which was restored by SSA treatment (Figure 3A) even in the shortest construct, −343/+25. These data demonstrate that the responsive element for SSA is located between −343 and +25 in the FLG promoter region.

Mutation of the AP1-binding site (mtAP1) in the −343/+25 construct abrogated the decrease in FLG promoter activity induced via TNFα + IFNγ, showing the recovery effect of SSA (Figure 3B) and suggesting that SSA restores reduced FLG transcription mediated via the AP1-binding site upon TNFα + IFNγ stimulation.

The EMSA data showed that the SSA treatment substantially reduced the DNA–protein complex induced by TNFα + IFNγ treatment (Figure 3C). These data demonstrate that the AP1-binding motif of the FLG promoter is the functional response element of SSA involved in the restoration of the reduced FLG promoter activity by TNFα + IFNγ.

### 2.4. SSA Inhibits TNFα + IFNγ-Induced Expression of c-Jun and FRA1

**Figure 4:**
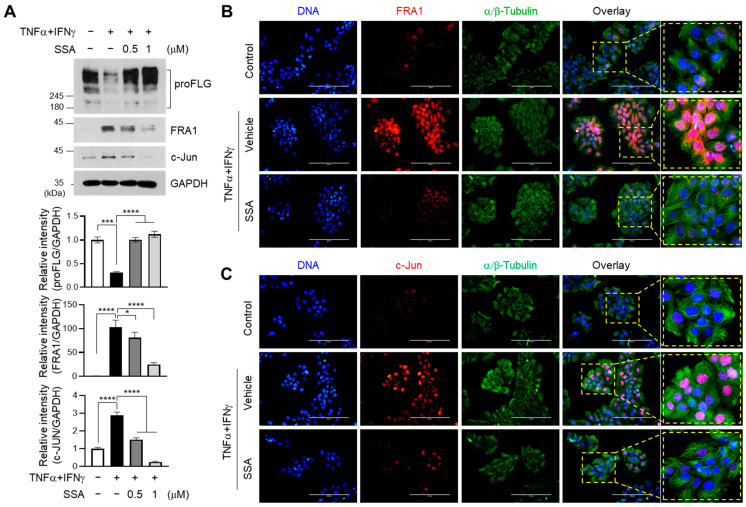
Effect of SSA on inhibiting the TNFα + IFNγ-induced expression of FRA1 and c-Jun. (**A**) HaCaT cells were treated with TNFα + IFNγ for 24 h with or without SSA. FLG, FRA1, c-Jun, and GAPDH protein levels were measured using immunoblotting. The quantitative band intensities of FLG, FRA1, and c-Jun proteins were normalized relative to that of GAPDH using ImageJ version 1.52a software. The data are displayed as mean ± SD (*n* = 3). * *p* < 0.1, *** *p* < 0.001, **** *p* < 0.0001. (**B**,**C**) HaCaT cells cultured on coverslips were treated with TNFα + IFNγ for 24 h in the presence or absence of 1 μM SSA, and immunofluorescence staining was performed using anti-FRA1 (**B**) and anti-c-JUN (**C**). Anti-α/β-Tubulin (green) antibodies were utilized to visualize the cytoplasm. Hoechst 33258 was utilized to counterstain the nuclear DNA (blue). Scale bars, 200 μm. SSA, saikosaponin A.

FRA-1 (fos-related antigen 1) and c-Jun are members of AP1 family [20]. FRA-1 is involved in cell proliferation, apoptosis, differentiation, inflammation, oncogenesis, and tumor metastasis, particularly in affecting inflammatory diseases [21]. Since TNFα + IFNγ activated the expression of FRA1 and c-Jun, which are recruited to the AP1-binding motif of the FLG promoter for the suppression of FLG expression [12], the impact of SSA on the modulation of FRA1 and c-Jun expressions was investigated. Treatment with SSA significantly (*p* < 0.001) restored FLG expression and suppressed the TNFα + IFNγ-induced expression of FRA1 and c-Jun in a dose-dependent manner (Figure 4A).

Fluorescence immunocytochemical staining showed that TNFα + IFNγ treatment induced the nuclear localization of FRA1 (Figure 4B) and c-Jun (Figure 4C), which was effectively reduced by SSA pretreatment. These data suggest that SSA treatment is associated with the downregulation of FRA1 and c-Jun expression upon restoration of FLG expression in TNFα + IFNγ-treated HaCaT cells.

### 2.5. SSA Abrogates TNFα + IFNγ-Induced Activation of ERK Signaling

**Figure 5:**
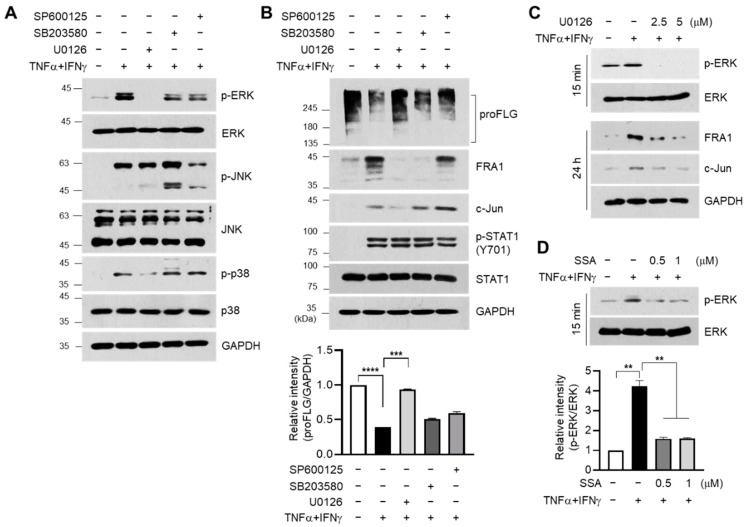
Effect of SSA on inhibiting TNFα + IFNγ-induced ERK activation. HaCaT cells were pretreated with U0126 (10 μM), SB203580 (20 μM), or SP600125 (25 μM) for 30 min, followed by exposure to 10 ng/mL TNFα + IFNγ. (**A**) After 15 min, whole-cell lysates were immunoblotted using antibodies against phospho-specific and total MAPK proteins. (**B**) The protein expression levels of FLG, FRA1, c-Jun, p-STAT1, STAT1, and GAPDH were measured using immunoblotting. (**C**) HaCaT cells were stimulated with TNFα + IFNγ (10 ng/mL) for 15 min or 24 h with or without U0126 (10 μM). Antibodies targeting phospho-ERK, total ERK, FRA1, c-Jun, and GAPDH were utilized in Western blotting. (**D**) HaCaT cells were stimulated with TNFα + IFNγ with or without SSA, followed by the assessment of p-ERK and ERK levels through immunoblotting. The intensity of FLG or p-ERK protein bands was normalized to GAPDH or total ERK proteins using ImageJ version 1.52a software. The data are displayed as mean ± SD (*n* = 3). ** *p* < 0.01, *** *p* < 0.001, **** *p* < 0.0001. SSA, saikosaponin A.

FRA1 and c-Jun were expressed when mitogen-activated protein kinases (MAPKs) were stably activated [22,23]. ERK activation induces FRA1 accumulation and dimerization of FRA1 and c-Jun at the AP1-binding site of target gene promoters [24,25]. As TNFα and IFNγ stimulate the MAPK pathway in HaCaT cells [13], the effect of SSA on the modulation of MAPK signaling was determined. Consistent with a previous report [13], the TNFα + IFNγ-induced phosphorylation of ERK1/2, JNK, and p38 kinase decreased upon pretreatment with U0126 (MAPK inhibitor), SB203580 (p38 kinase inhibitor), and SP600125 (JNK inhibitor) (Figure 5A). The effect of these inhibitors on the modulation of FLG, FRA1, and c-Jun expressions was further addressed under TNFα + IFNγ stimulation. Immunoblotting data showed that U0126 pretreatment restored the suppression of FLG expression and repressed the expression of FRA1 and c-Jun in TNFα + IFNγ-treated HaCaT keratinocytes (Figure 5B). Furthermore, the inhibition of ERK phosphorylation by U0126 pretreatment suppressed the accumulation of FRA1 and c-Jun proteins induced by TNFα + IFNγ (Figure 5C). The effect of SSA on ERK phosphorylation was determined. SSA reduced the TNFα + IFNγ-induced phosphorylation of ERK1 in a dose-dependent manner (Figure 5D). These data suggest that SSA modulates the TNFα + IFNγ-induced expression of FLG, FRA1, and c-Jun via ERK inhibition.

### 2.6. SSA Inhibits the Binding of c-Jun and FRA1 to the AP1 Motif of the FLG Promoter under TNFα + IFNγ Stimulation

**Figure 6:**
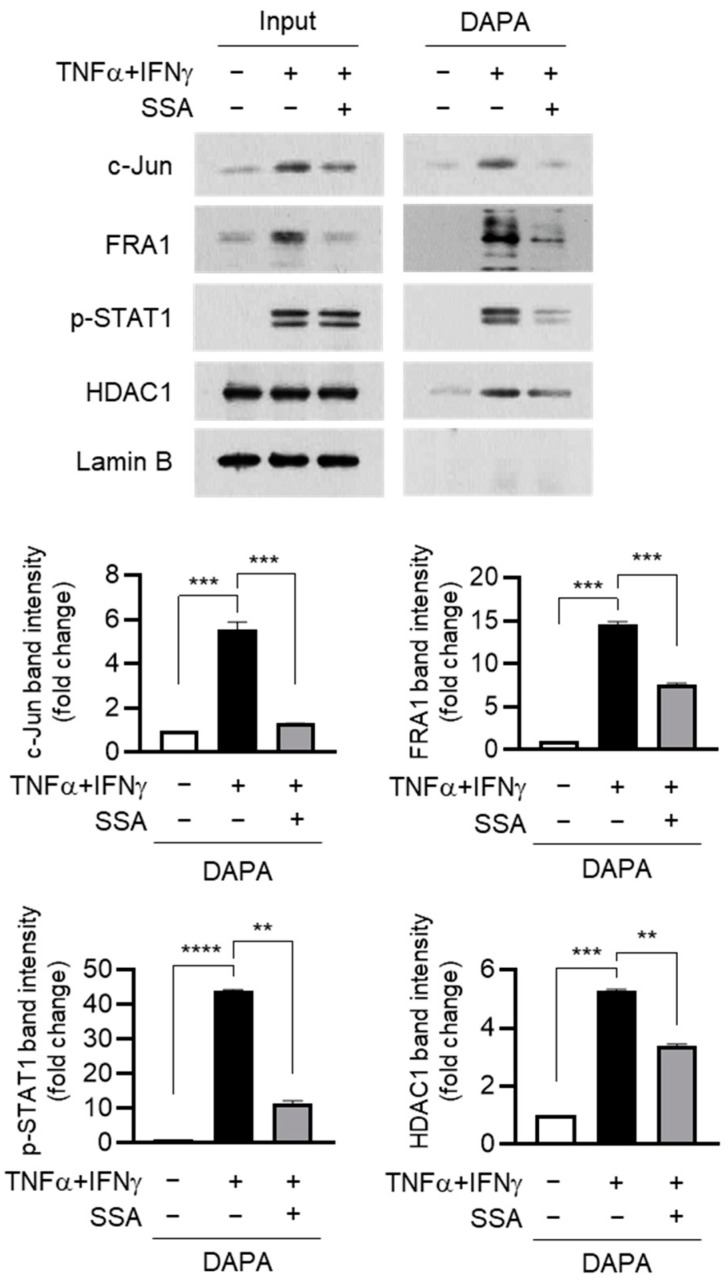
Effect of SSA on inhibiting the binding of c-Jun and FRA1 to the AP1 motif in the FLG promoter. Nuclear extracts from HaCaT cells exposed to TNFα + IFNγ (10 ng/mL) for 24 h were incubated with biotin-labeled oligonucleotide probes encoding AP1-binding sequences in the FLG promoter, followed by precipitation with streptavidin-conjugated agarose beads. Co-precipitated proteins were analyzed via immunoblotting using specific antibodies against c-Jun, FRA1, p-STAT1 (Y701), and HDAC1. Lamin B was used as a nuclear marker. The relative band intensities of c-Jun and FRA1 proteins were quantified using ImageJ version 1.52a software. The data are displayed as mean ± SD (*n* = 3). ** *p* < 0.01, *** *p* < 0.001, **** *p* < 0.0001. DAPA, DNA affinity precipitation assay; SSA, saikosaponin A.

TNFα + IFNγ stimulation suppresses FLG expression by promoting the FRA1:c-JUN:HDAC1:p-STAT1 complex, and IFNγ-induced p-STAT1 was required to recruit HDAC1 to the repressor complex [12]. The effect of SSA on the DNA binding affinity of these proteins was determined using a DNA affinity precipitation assay (DAPA). Pretreatment with SSA decreased the expression of FRA1 and c-Jun in the nucleus (input), thereby reducing the DNA binding of FRA1, c-Jun, p-STAT1, and HDAC1 (Figure 6). These findings suggest that SSA restores FLG expression by repressing the expression of FRA1 and c-Jun, thus preventing the recruitment of the repressor complex to the FLG promoter.

**Figure 7:**
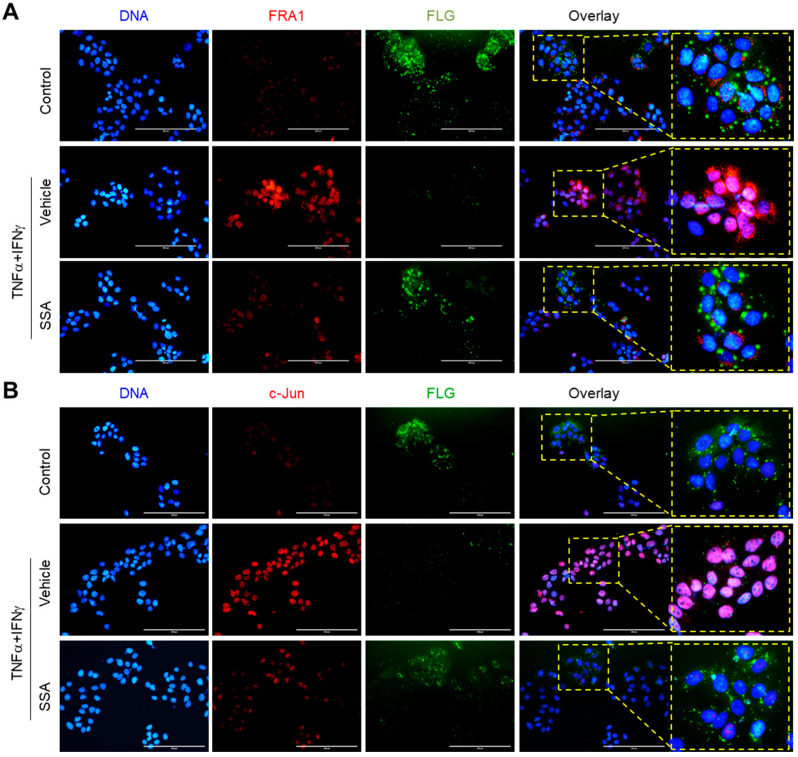
Subcellular localization of the TNFα + IFNγ-induced expression of FRA1, c-Jun, and FLG. HaCaT cells cultured on coverslips were treated with 10 ng/mL TNFα + IFNγ for 24 h with 1 μM SSA. Immunofluorescence staining showed the localization of FRA1 (red) and FLG (green) (**A**) or c-Jun (red) and FLG (green) (**B**). Hoechst 33258 was utilized to counterstain the nuclear DNA (blue). Scale bars, 200 μm. DNCB, 2,4-dinitrochlorobenzene; p-ERK, phosphorylated ERK; SSA, saikosaponin A.

The fluorescent immunocytochemical staining showed that SSA reduced the TNFα + IFNγ-induced nuclear localization of FRA1 (Figure 7A) and c-Jun (Figure 7B), along with the restoration of FLG expression. Therefore, we propose that SSA restores FLG expression by reducing the levels of FRA1 and c-Jun in the nucleus.

### 2.7. Effect of Topical Application of SSA on the Amelioration of DNCB-Induced AD-Like Skin Lesions in BALB/c Mice

**Figure 8:**
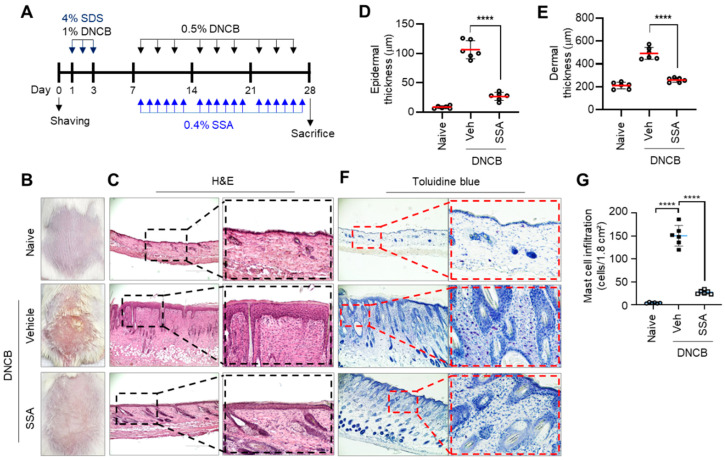
Effect of SSA on the amelioration of DNCB-induced AD-like skin lesions in BALB/c mice. (**A**) Experimental schedule to induce atopic dermatitis (AD)-like skin lesions. DNCB, dinitrochlorobenzene. (**B**) Representative images of the back skin of BALB/c mice photographed on day 28: naïve, DNCB + vehicle (70% ethanol); DNCB + SSA (0.4%). (**C**) H&E, hematoxylin and eosin staining. Epidermal thickness (**D**) and dermal thickness (**E**) were measured using ImageJ version 1.52a software. The infiltration of inflammatory cells was analyzed using toluidine blue staining (**F**). The number of permeabilized cells was quantified graphically by counting per area (**G**). Scale bars, 200 μm. The data are presented in the form of mean ± SD (*n* = 6). **** *p* < 0.0001. SDS, sodium dodecyl sulfate; DNCB, 2,4-dinitrochlorobenzene; SSA, saikosaponin A; Veh, vehicle.

BALB/c mice were repeatedly challenged with a DNCB solution to induce AD-like skin lesions on the back skin (Figure 8A). DNCB-induced AD-like phenotypes were improved by topically applying SSA (Figure 8B). H&E staining showed that SSA application significantly (*p* < 0.001) reduced the DNCB-induced thickening of the epidermis and dermis (Figure 8C), as evaluated by morphometric analysis (Figure 8D,E). In chronic skin inflammation, immune cells, such as Th lymphocytes, eosinophils, and mast cells, infiltrate the dermis and epidermis. Toluidine blue (TB) staining showed the infiltration of immune cells in DNCB-treated dorsal skin; however, the topical application of SSA substantially reduced the number of infiltrating immune cells (*p* < 0.001) (Figure 8F). These data demonstrated that SSA improved AD-like skin inflammation in DNCB-challenged BALB/c mice.

### 2.8. SSA Modulates the Expression of FLG, FRA1, c-Jun, and p-ERK in DNCB-Treated BALB/c Mice

**Figure 9:**
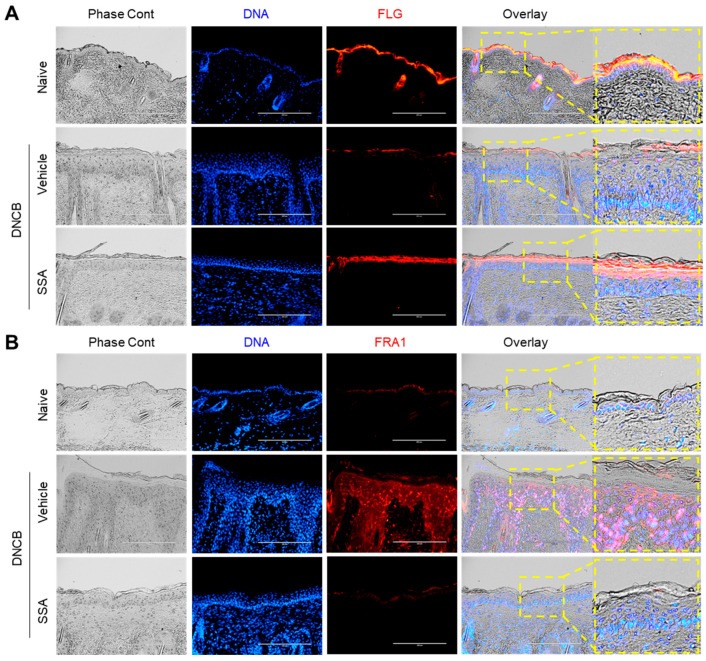
Effect of SSA on restoring the changed expressions of FLG and FRA1 in DNCB-challenged BALB/c mice. BALB/c mice were either treated with DNCB + 70% ethanol (vehicle) or DNCB + SSA (0.4%). Paraffin-embedded skin tissue sections were deparaffinized and subjected to immunofluorescence staining with antibodies against FLG (**A**) and FRA1 (**B**). Secondary antibodies conjugated with Alexa Fluor 488 (green) or rhodamine red-X (red) were employed. Hoechst 33258 was utilized to counterstain nuclear DNA (blue). Scale bars represent 200 μm. DNCB, 2,4-dinitrochlorobenzene; FLG, filaggrin; FRA1, fos-related antigen 1; SSA, saikosaponin A.

**Figure 10:**
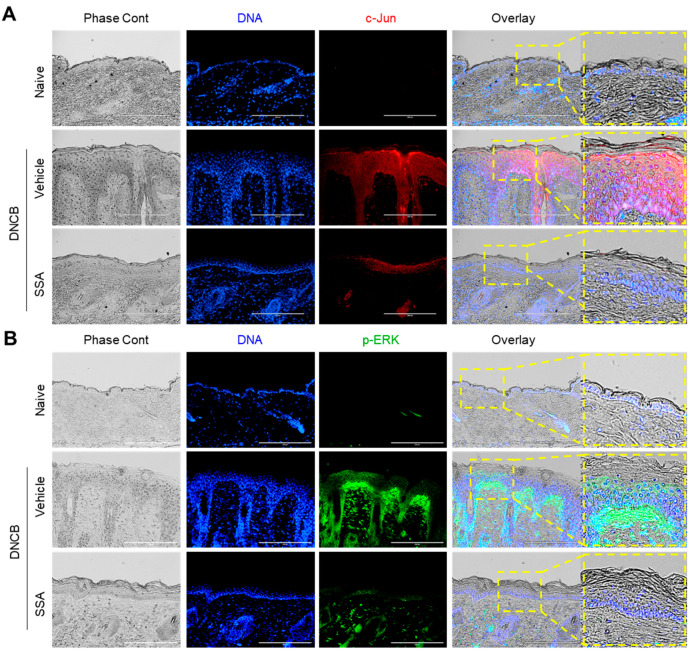
Effect of SSA on restoring the changed expressions of c-Jun and p-ERK in DNCB-challenged BALB/c mice. BALB/c mice were either treated with DNCB + 70% ethanol (vehicle) or DNCB + SSA (0.4%). Paraffin-embedded skin tissue sections were deparaffinized and subjected to immunofluorescence staining with antibodies against c-Jun (**A**) and p-ERK (**B**). Secondary antibodies conjugated with Alexa Fluor 488 (green) or rhodamine red-X (red) were employed. Hoechst 33258 was utilized to counterstain the nuclear DNA (blue). Scale bars represent 200 μm. DNCB, 2,4-dinitrochlorobenzene; p-ERK, phosphorylated ERK; SSA, saikosaponin A.

Suppression of FLG expression is a well-documented feature in DNCB-induced AD-like mouse skin [12,14]; hence, the FLG reduction was examined in AD-like skin lesions by immunofluorescence staining. FLG expression was reduced in the DNCB-challenged group, which was rescued by the topical application of SSA (Figure 9A). The expressions of FRA1 (Figure 9B), c-Jun (Figure 10A), and p-ERK (Figure 10B) were intensified in the DNCB-treated dorsal skin compared with that in the vehicle-treated group. Conversely, SSA significantly reduced the stained forms of these proteins. These results demonstrate that SSA effectively recovers impaired FLG expression in vivo in DNCB-induced AD-like skin inflammation by suppressing FRA1, c-Jun, and p-ERK expression.

## 3. Discussion

FLG is an essential barrier protein that protects the body from invading pathogens. The abnormal expression of FLG is associated with disruption of the skin barrier and pathogenesis of inflammatory skin diseases, such as AD and psoriasis [9]. Several saikosaponins, including SSA, isolated from a Radix Bupleuri extract, demonstrate a range of pharmacological properties, such as antiviral, anticancer, and anti-inflammatory activities [17,18,26]. Indeed, SSA ameliorates AD-like skin inflammation by reducing EGR1-mediated TSLP expression in HaCaT keratinocytes [13]; however, the effect of SSA on the expression of FLG remains unclear.

This study found that SSA treatment effectively counteracts the inhibitory effects on FLG expression induced by TNFα + IFNγ stimulation at the transcriptional level. FLG transcription is regulated by diverse transcription factors, including AP1 and STAT3 [14,27]. Since the AP1-binding motif within the FLG promoter is crucial in mediating the suppression of FLG expression during TNFα + IFNγ treatment [12], this study looked into how SSA affects the regulation of FLG transcription through the AP1 motif and the decrease in FRA1 and c-Jun expression in the nucleus caused by TNFα + IFNγ stimulation in keratinocytes. Stable expression of FRA1 and c-Jun was observed when the MAPK pathway was activated in cells [24]. The pre-incubation of cells with inhibitors targeting ERK1/2, JNK1/2, and p-p38 kinase prior to TNFα + IFNγ exposure revealed that only the inhibition of ERK1/2 reversed the decrease in FLG expression by preventing the accumulation of FRA1 and c-Jun. In addition, SSA inhibited ERK1/2 phosphorylation, suggesting that SSA reduced the expression of FRA1 and c-Jun by abrogating ERK1/2 activation. However, in this study, the inhibitory mechanism of SSA on ERK activation is not elucidated; therefore, further studies are required to address this question.

Previously, it has been demonstrated that in MCF7 breast cancer cells, STAT3 collaborates with FRA1 and c-JUN AP1 components to activate the MMP9 promoter [28], while in HepG2 hepatoma cells, STAT3 associates with HDAC1 to inhibit IL6-induced CCL2 transcription [29]. Furthermore, the activation of STAT1 by IFNγ leads to the binding of STAT1 with HDAC1 [30]. Given that class I HDACs play a crucial role in maintaining skin homeostasis [31] and that STAT1 activity is heightened in inflamed skin [32], it is suggested that IFNγ-activated STAT1 might also interact with FRA1:c-JUN, potentially recruiting HDAC1 to the FLG promoter in keratinocytes. In this study, the impact of SSA on the recruitment of the repressor complex was investigated. The results showed that SSA specifically reduced the expression of FRA1 and c-Jun induced by TNFα but did not affect the IFNγ-induced STAT1 phosphorylation. However, when TNFα and IFNγ were combined, SSA inhibited the recruitment of other co-repressor proteins to the AP1 motif of the FLG promoter by reducing the expression of FRA1 and c-Jun. Immunofluorescence staining confirmed the correlation between the nuclear expression of FRA1 and the expression of c-Jun and FLG. Furthermore, the topical application of SSA improved AD-like skin lesions caused by DNCB in BALB/c mice. Moreover, the upregulation of FLG and the downregulation of FRA1, c-Jun, and p-ERK1/2 were observed in skin tissues from mice exposed to DNCB or DNCB + SSA, demonstrating that SSA effectively restores FLG expression in vivo in inflamed skin regions.

While SSA has various pharmacological properties, including anti-inflammatory and antiviral effects, its use must be carefully monitored due to its toxicity at higher doses or with prolonged use [33]. While most of the research on SSA toxicity has focused on its systemic administration (oral or injection), topical application of SSA remains well-known to have the potential to cause toxicity at both local and systemic levels. Therefore, further research is needed to understand better its safety profile, optimal dosing, and mechanisms of toxicity in clinical settings.

Although animal models provide valuable insights into the pathophysiology, immunology, and potential therapeutic interventions for AD, moving from animal models to human trials in treating AD involves several limitations and challenges. Human skin is structurally and functionally different from commonly used animal models [34]. Also, the human immune system differs from mouse models regarding cell types, cytokine profiles, and receptor expression. The pharmacokinetics and pharmacodynamics of drugs can differ significantly between mouse models and humans. Variations in skin permeability, metabolism, and systemic absorption rates can lead to different drug efficacy and safety profiles [35]. These limitations highlight the importance of developing more predictive models, such as humanized animal models and advanced human skin models [36].

A major shortcoming of the present work is the lack of detailed information about the presence of the co-repressor complex at the AP1 site in the FLG gene promoter, mainly due to difficulties in the experimental methodology. Since HDAC1 is known to form complexes with various repressor proteins such as SIN3 or NuRD [37,38], additional experiments are required to determine the specific co-repressor proteins involved in the formation of the complex with FRA1:c-JUN:HDAC1 for the suppression of FLG expression in response to SSA treatment.

## 4. Materials and Methods

### 4.1. Materials

Saikosaponin-A (SSA) was bought from TCI Chemicals (Tokyo, Japan). TNFα and IFNγ were purchased from ProSpec-Tany TechnoGene Ltd. (Ness Ziona, Israel). The Firefly luciferase assay system was obtained from Promega (Madison, WI, USA). The analysis of protein-binding sites in the gene promoter region was conducted using MatInspector, a web tool available at https://scicrunch.org/resolver/SCR_008036/ (accessed on 1 March 2023). Antibodies to FLG for immunofluorescence staining were purchased from BioLegend (San Diego, CA, USA). Antibodies to phospho-extracellular signal-regulated kinase (ERK)1/2 (Thr202/Tyr204), phospho-p38 (Thr180/Tyr182), phospho-JNK1/2 (Thr183/Tyr185), histone deacetylase 1 (HDAC1), and phospho-STAT1 (Tyr701) were from Cell Signaling Technology (Danvers, MA, USA). Antibodies to FLG, fos-like antigen protein 1 (FRA1), c-JUN, and glyceraldehyde 3-phosphate dehydrogenase (GAPDH) for immunoblotting were from Santa Cruz Biotechnology (Dallas, TX, USA). Secondary antibodies conjugated to Alexa Fluor 488 or rhodamine red X were purchased from Jackson ImmunoResearch Laboratories (West Grove, PA, USA). Other substances were purchased from Sigma-Aldrich (St. Louis, MO, USA).

### 4.2. Cells and Culture Conditions

HaCaT cells obtained from Cell Lines Service (Eppelheim, Germany) were maintained in a medium with fetal bovine serum from HyClone (Logan, UT, USA), along with 0.5% penicillin-streptomycin from Sigma-Aldrich.

### 4.3. Cytotoxicity Assay

The cellular viability was assessed using a Cell Counting Kit-8 (Dojindo Molecular Technologies, Rockville, MD, USA) as described previously [39]. The formation of orange-colored WST-8 formazan dye produced by the reduction in WST-8 in living cells was quantitated at an absorbance of 450 nm using an Emax Endpoint ELISA Microplate Reader (Molecular Devices, Sunnyvale, CA, USA).

### 4.4. Reverse Transcription Polymerase Chain Reaction and Quantitative Real-Time PCR

Total RNA was extracted using the TRIzol RNA extraction kit (Invitrogen, Carlsbad, CA, USA). The synthesis of cDNA and the reverse transcription polymerase chain (RT-PCR) reaction were performed as described previously [40]. Gene-specific PCR primers were as follows: FLG forward, 5′-CAAATCCTGAAG AATCCAGATGAC-3′; FLG reverse, 5′-TGCTTGAGCCAACTTGAATACC-3′; GAPDH forward, 5′-CCAAGGAGTAAGAAACCCTGGAC-3′; GAPDH reverse, 5′-GGGCCGAGT TGGGATAGGG-3′.

qPCR primers and SYBR green-based fluorescent probes specific for FLG (id: qHsaCEP0039328) and GAPDH (id: qHsaCEP0041396) were purchased from Bio-Rad. The FLG mRNA expression was normalized to GAPDH by utilizing the software provided by the manufacturer (Bio-Rad, Hercules, CA, USA).

### 4.5. Immunoblot Analysis

Keratinocytes were lysed, and an immunoblot analysis was performed as described previously [41]. The relative protein band intensities were measured using ImageJ version 1.52a software and compared to GAPDH levels.

### 4.6. Immunofluorescence

HaCaT cells cultured on coverslips were exposed to either vehicle (phosphate-buffered saline; PBS) or 10 ng/mL TNFα + IFNγ for 24 h then fixed, permeabilized, and subjected to immunofluorescence as described previously [12]. Fluorescent cells were examined under an EVOS FL Fluorescence Microscope (Advanced Microscopy Group, Bothell, WA, USA).

### 4.7. Construction and Internal Deletion of Human FLG Promoter–Reporter Constructs

Serial deletion constructs of human FLG promoter fragments and site-specific mutation of the AP1-binding site (mtAP1) were described elsewhere [12].

### 4.8. Luciferase Promoter–Reporter Assay

HaCaT cells cultured in 12-well plates were transiently transfected with 0.2 μg FLG promoter constructs and exposed to TNFα (10 ng/mL) + IFNγ (10 ng/mL). The Dual-Glo luciferase assay system (Promega) was utilized to quantify the activity of firefly luciferase with a dual luminometer (Centro LB960; Berthold Tech, Bad Wildbad, Germany) as described previously [12]. The luciferase activity of untreated cells was set to 1.

### 4.9. Electrophoretic Mobility Shift Assay

Protein–DNA binding ability was verified using a LightShift Chemiluminescent EMSA Kit (Thermo Fisher Scientific, Waltham, MA, USA). A biotinylated deoxy-oligonucleotide probe corresponding to the AP1-binding sequence was synthesized by Macrogen (5′-biotin-GGT TAG GAA TGA ATC AGA CCA TCC C-3′). The Nuclear and Cytoplasmic Extraction Kit from Thermo Fisher Scientific was employed to extract nuclear proteins. The detection of DNA–protein complexes was performed using an Amersham ECL Western Blotting Detection Kit (GE Healthcare Life Science, Chicago, IL, USA) as described previously [12].

### 4.10. DNA Affinity Precipitation Assay

Nuclear extracts were prepared from HaCaT cells exposed to 10 ng/mL TNFα + IFNγ, with a NE-PER Nuclear and Cytoplasmic Extraction Reagent Kit (Thermo Fisher Scientific). Nuclear proteins (40 μg) were incubated overnight with 200 pmol of biotin-AP1 oligonucleotides (5′-biotin-GGTTAGGAATGAATCAGACCATCCC-3′) and streptavidin-conjugated agarose beads (Invitrogen). The beads were collected using a tabletop centrifuge. After washing with PBS, the proteins were eluted from the beads by boiling with 2× Laemmli sample buffer, and immunoblotting was performed using antibodies against FRA1, c-Jun, p-STAT1, HDAC1, and the Lamin B nuclear marker protein as previously described [12].

### 4.11. DNCB-Challenged Atopic-Dermatitis-like Skin Lesions in BALB/c Mice

BALB/c mice (seven-week-old male) were purchased from Orient Bio, Inc. (Seongnam, Republic of Korea). The mice were divided into four groups in a random manner (*n* = 6 in each group): Group I, vehicle (70% ethanol); Group II, 0.5% DNCB; and Group III, 0.5% DNCB + 0.4% SSA. All mice were sensitized with 4% SDS on the back skin to disrupt the skin barrier, and 0.5% DNCB in an acetone: olive oil mixture (1:3, *v*/*v*) with or without 0.4% SSA was applied as described previously [13]. On day 28, all mice were euthanized, and tissue sections were prepared.

### 4.12. Fluorescent Immunohistochemical Staining

Paraffin-embedded skin tissues underwent deparaffinization and hydration, followed by incubation with blocking buffer as described previously [12]. Fluorescent immunohistochemical staining was carried out with primary antibodies against FLG (1:100 dilution), FRA1 (1:100 dilution), and c-Jun (1:200 dilution), followed by secondary antibodies conjugated with rhodamine Red-X (Jackson ImmunoResearch Laboratories, 1:300 dilution). Nuclear DNA was counterstained with a Hoechst 33258 solution and coverslipped with a fluorescence mounting medium (ProLong Gold Antifade Reagent; Invitrogen). Images were taken using an EVOS FL fluorescence microscope (Advanced Microscopy Group, Bothell, WA, USA).

### 4.13. Statistical Analyses

The data were analyzed using one-way analysis of variance (ANOVA) followed by Sidak’s multiple comparison test with GraphPad Prism version 8.4.2 (GraphPad Software, Inc., La Jolla, CA, USA). For all analyses, a *p*-value less than 0.05 indicated a statistically significant difference.

## 5. Conclusions

Our findings demonstrate that SSA effectively restores impaired FLG expression by reducing ERK-mediated FRA1 and c-Jun expression under TNFα + IFNγ stimulation. Previously, it has been reported that SSA suppresses TNFα-induced TSLP expression in keratinocytes. This study further highlights the anti-inflammatory effect of SSA on AD-like skin inflammation, suggesting the potential of SSA as a promising therapeutic agent for treating atopic dermatitis. However, additional studies are required to explore its human clinical efficacy.

## Data Availability

The data presented in this study are available on request from the corresponding author.
